# Supplementary material for: Molecular Detection and Characterization of Borrelia garinii (Spirochaetales: Borreliaceae) in Ixodes nipponensis (Ixodida: Ixodidae) Parasitizing a Dog in Korea
Source: Pathogens. 2019 Dec 6;8(4):289. doi: 10.3390/pathogens8040289 (PMC6963618; doi:10.3390/pathogens8040289)
Supplement: Supplementary file 1 [file pathogens-08-00289-s001.zip › pathogens-653875-supplementary/pathogens-653875-Supplementary Figures.pdf]

Supplementary Figures

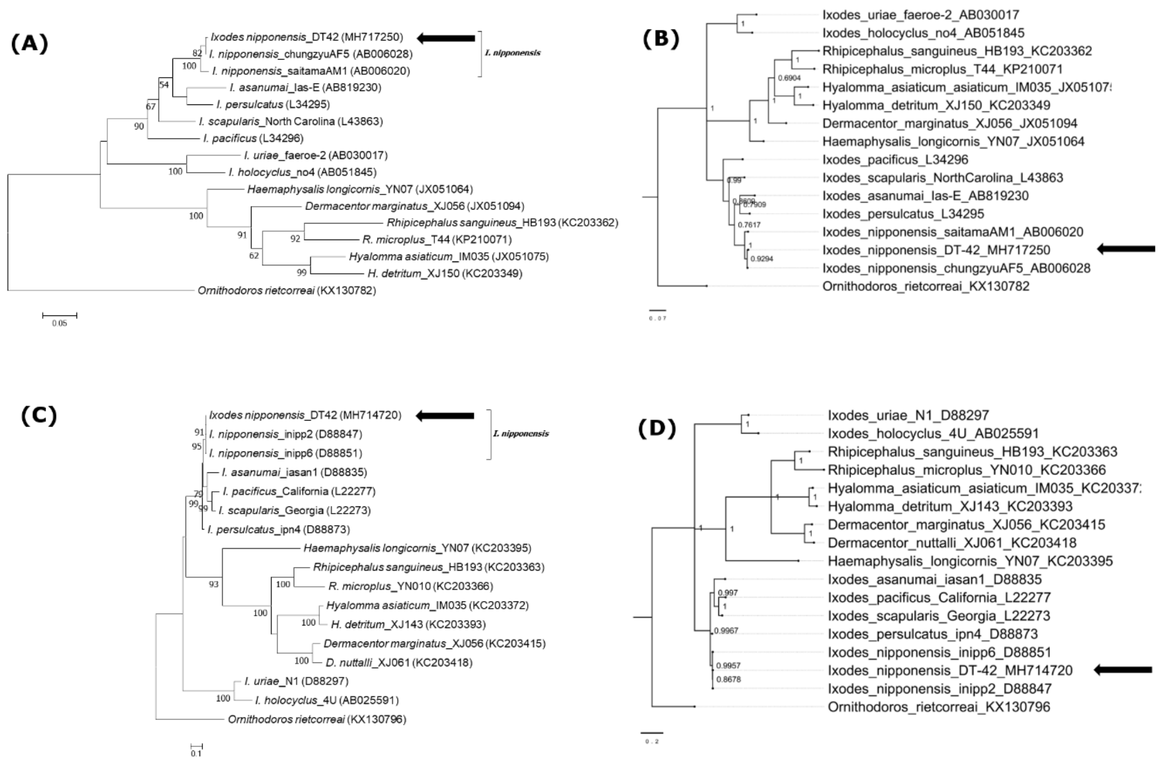

Figure S1. Phylogenetic analysis of tick species based on (A and B) mitochondrial 16S rRNA and (C and D) the second intergenic spacer region. The trees (A and C) are constructed based on the maximum-likelihood method with 500 bootstrap replications using MEGA 7.0. Bootstrap values less than 50 are omitted. The trees (B and D) are constructed based on the Bayesian inference method using MrBayes v3.2.6 and are visualised using FigTree v1.4.3. Posterior probability values are shown in the nodes. The sequence identified in this study is indicated by arrows.

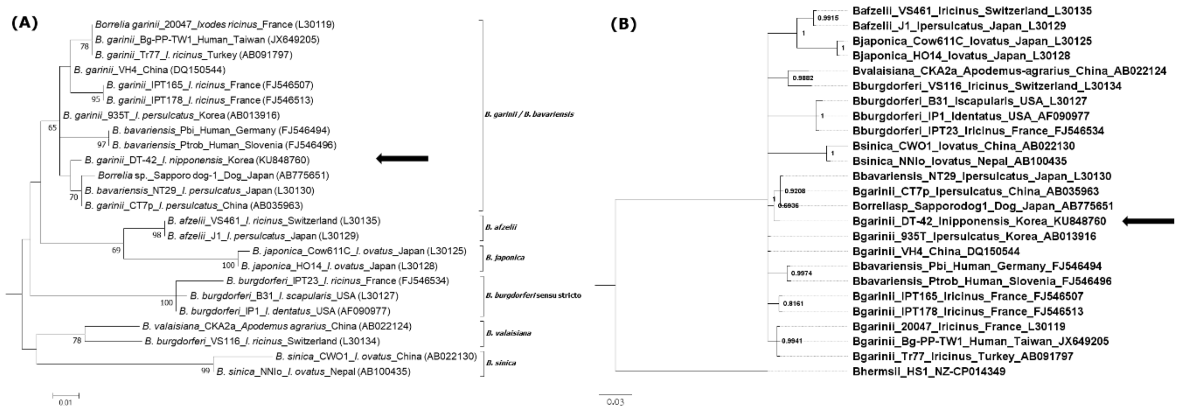

Figure S2. Phylogenetic analysis of *Borrelia garinii* based on the 5S–23S intergenic spacer region. The tree (A) is constructed based on the maximum-likelihood method with 500 bootstrap replications using MEGA 7.0. Bootstrap values less than 50 are omitted. The tree (B) is constructed based on the Bayesian inference method using MrBayes v3.2.6 and is visualised using FigTree v1.4.3. Posterior probability values are shown in the nodes. *Borrelia* spp., isolate, host, identified country, and the nucleotide accession number are described in the tree. The sequence identified in this study is indicated by an arrow.

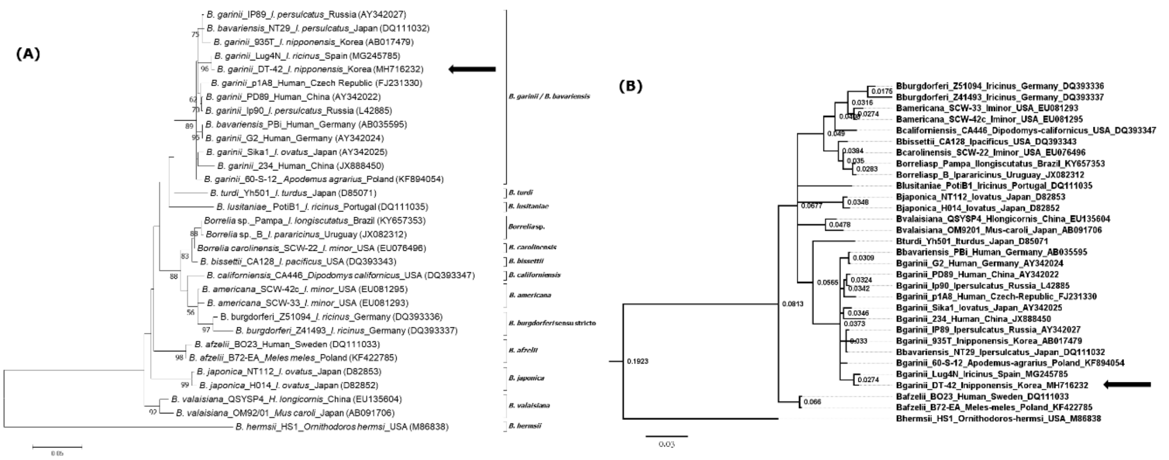

Figure S3. Phylogenetic analysis of *Borrelia garinii* based on the *flagellin* gene. The tree (A) is constructed based on the maximum-likelihood method with 500 bootstrap replications using MEGA 7.0. Bootstrap values less than 50 are omitted. The tree (B) is constructed based on the Bayesian inference method using MrBayes v3.2.6 and is visualised using FigTree v1.4.3. Posterior probability values are shown in the nodes. *Borrelia* spp., isolate, host, identified country, and the nucleotide accession number are described in the tree. The sequence identified in this study is indicated by an arrow.

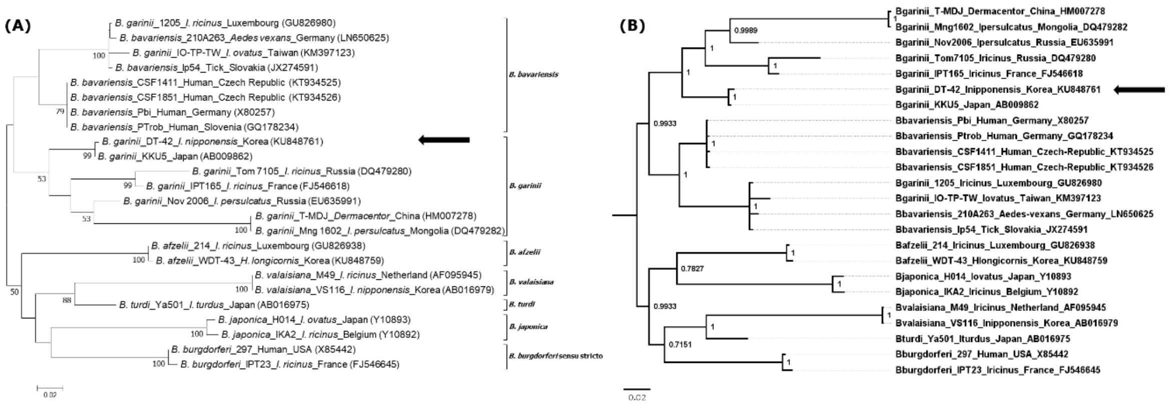

Figure S4. Phylogenetic analysis of *Borrelia garinii* based on the outer surface protein A (*ospA*) gene. The tree (A) is constructed based on the maximum-likelihood method with 500 bootstrap replications using MEGA 7.0. Bootstrap values less than 50 are omitted. The tree (B) is constructed based on the Bayesian inference method using MrBayes v3.2.6 and is visualised using FigTree v1.4.3. Posterior probability values are shown in the nodes. *Borrelia* spp., isolate, host, identified country, and the nucleotide accession number are described in the tree. The sequence identified in this study is indicated by an arrow.
