# Supplementary material for: Molecular Detection and Characterization of Borrelia garinii (Spirochaetales: Borreliaceae) in Ixodes nipponensis (Ixodida: Ixodidae) Parasitizing a Dog in Korea
Source: Pathogens. 2019 Dec 6;8(4):289. doi: 10.3390/pathogens8040289 (PMC6963618; doi:10.3390/pathogens8040289)
Supplement: Supplementary file 1 [file pathogens-08-00289-s001.zip › pathogens-653875-supplementary/pathogens-653875-Supplementary Tables.pdf]

Table S1. Mitochondrial 16S rRNA sequences from ticks included in the phylogenetic analysis summarized according to species, strain or isolate, length (bp), and GenBank accession number.

	Species	Strain or isolate	length (bp)	GenBank accession no.
1	*Ixodes nipponensis*	DT-42	343	MH717250
2	*Ixodes nipponensis*	chungzyuAF5	454	AB006028
3	*Ixodes nipponensis*	saitamaAM1	456	AB006020
4	*Ixodes asanumai*	Ias-E	412	AB819230
5	*Ixodes persulcatus*	N/A	457	L34295
6	*Ixodes scapularis*	North Carolina	456	L43863
7	*Ixodes pacificus*	N/A	459	L64296
8	*Ixodes uriae*	faeroe-2	457	AB030017
9	*Ixodes holocyclus*	no4	464	AB051845
10	*Haemaphysalis longicornis*	YN07	455	JX051064
11	*Dermacentor marginatus*	XJ056	451	JX051094
12	*Rhipicephalus sanguineus*	HB193	430	KC203362
13	*Rhipicephalus microplus*	T44	427	KP210071
14	*Hyalomma asiaticum*	IM035	445	JX051075
15	*Hyalomma detritum*	XJ150	447	KC203349
16	*Ornithodoros rietcorreai*	N/A	427	KX130782

Table S2. The second intergenic spacer region sequences from ticks included in the phylogenetic analysis summarized according to species, strain or isolate, length (bp), and GenBank accession number.

	Species	Strain or isolate	length (bp)	GenBank accession no.
1	*Ixodes nipponensis*	DT-42	769	MH714720
2	*Ixodes nipponensis*	inipp6	830	D88851
3	*Ixodes nipponensis*	inipp2	830	D88847
4	*Ixodes persulcatus*	ipn4	824	D88873
5	*Ixodes pacificus*	California	1247	L22277
6	*Ixodes scapularis*	Georgia	1208	L22273
7	*Ixodes asanumai*	iasan1	817	D88835
8	*Haemaphysalis longicornis*	YN07	1592	KC203395
9	*Rhipicephalus sanguineus*	HB193	1084	KC203363
10	*Rhipicephalus microplus*	YN010	1098	KC203366
11	*Hyalomma asiaticum*	IM035	1418	KC203372
12	*Hyalomma detritum*	XJ143	1369	KC203393
13	*Dermacentor marginatus*	XJ056	1063	KC203415
14	*Dermacentor nuttalli*	XJ061	1059	KC203418
15	*Ixodes uriae*	N1	905	D88297
16	*Ixodes holocyclus*	4U	793	AB025591
17	*Ornithodoros rietcorreai*	N/A	434	KX130796

Table S3. The 5S-23S intergenic spacer region of *Borrelia* species included in the phylogenetic analysis summarized according to species, strain or isolate, length (bp), and GenBank accession number.

	Species	Strain or isolate	Host	Country	length (bp)	GenBank accession no.
1	*B. garinii*	DT-42	*Ixodes nipponensis*	Korea	255	KU848760
2	*B. afzelii*	J1	*Ixodes persulcatus*	Japan	246	L30129
3	*B. afzelii*	VS461	*Ixodes ricinus*	Switzerland	246	L30135
4	*B. bavariensis*	Ptrob	Human	Slovenia	163	FJ546496
5	*B. burgdorferi*	IP1	*Ixodes dentatus*	USA	238	AF090977
6	*B. burgdorferi*	B31	*Ixodes scapularis*	USA	254	L30127
7	*B. burgdorferi*	IPT23	*Ixodes ricinus*	France	164	FJ546534
8	*B. garinii*	935T	*Ixodes persulcatus*	Korea	237	AB013916
9	*B. garinii*	CT7p	*Ixodes persulcatus*	China	253	AB035963
10	*B. garinii*	Sapporo dog-1	Dog	Japan	199	AB775651
11	*B. garinii*	Bg-PP-TW1	human skin	Taiwan	221	JX649205
12	*B. garinii*	20047	*Ixodes ricinus*	France	253	L30119
13	*B. garinii*	NT29	*Ixodes persulcatus*	Japan	253	L30130
14	*B. garinii*	Pbi	Human	Germany	163	FJ546494
15	*B. garinii*	VH4	N/A	China	255	DQ150544
16	*B. garinii*	Tr77	*Ixodes ricinus*	Turkey	253	AB091797
17	*B. garinii*	IPT165	*Ixodes ricinus*	France	163	FJ546507
18	*B. garinii*	IPT178	*Ixodes ricinus*	France	163	FJ546513
19	*B. japonica*	Cow611C	*Ixodes ovatus*	Japan	236	L30125
20	*B. japonica*	HO14	*Ixodes ovatus*	Japan	236	L30128
21	*B. sinica*	CWO1	*Ixodes ovatus*	China	236	AB022130
22	*B. sinica*	NNIo	*Ixodes ovatus*	Nepal	238	AB100435
23	*B. valaisiana*	CKA2a	*Apodemus agrarius*	China	254	AB022124

24	*B. valaisiana*	VS116	*Ixodes ricinus*	Switzerland	255	L30134

Table S4. *Flagellin* gene of *Borrelia* species included in the phylogenetic analysis summarized according to species, strain or isolate, length (bp), and GenBank accession number.

	Species	Strain or isolate	Host	Country	length (bp)	GenBank accession no.
1	*B. garinii*	DT-42	*Ixodes nipponensis*	Korea	354	MH716232
2	*B. afzelii*	BO23	Human	Sweden	456	DQ111033
3	*B. afzelii*	B72-EA	*Meles meles*	Poland	789	KF422785
4	*B. americana*	SCW-33	*Ixodes minor*	USA	497	EU081293
5	*B. americana*	SCW-42c	*Ixodes minor*	USA	497	EU081295
6	*B. bissettii*	CA128	*Ixodes pacificus*	USA	456	DQ393343
7	*B. burgdorferi*	Z51094	*Ixodes ricinus*	Germany	459	DQ393336
8	*B. burgdorferi*	Z41493	*Ixodes ricinus*	Germany	456	DQ393337
9	*B. californiensis*	CA446	*Dipodomys californicus*	USA	456	DQ393347
10	*B. carolinensis*	SCW-22	*Ixodes minor*	USA	497	EU076496
11	*B. garinii*	935T	*Ixodes nipponensis*	Korea	952	AB017479
12	*B. garinii*	Pbi	Human	Germany	600	AB035595
13	*B. garinii*	PD89	Human	China	584	AY342022
14	*B. garinii*	G2	Human	Germany	584	AY342024
15	*B. garinii*	SIKA1	*Ixodes ovatus*	Japan	584	AY342025
16	*B. garinii*	IP89	*Ixodes persulcatus*	Russia	584	AY342027
17	*B. garinii*	NT29	*Ixodes persulcatus*	Japan	456	DQ111032
18	*B. garinii*	p1A8	Human	Czech Republic	495	FJ231330
19	*B. garinii*	234	Human	China	579	JX888450
20	*B. garinii*	60-S-12	*Apodemus agrarius*	Poland	587	KF894054
21	*B. garinii*	lp90	*Ixodes persulcatus*	Russia	1011	L42885
22	*B. garinii*	Lug4N	*Ixodes ricinus*	Spain	359	MG245785
23	*B. hermsii*	HS1	*Ornithodoros hermsi*	USA	1736	M86838
24	*B. japonica*	H014	*Ixodes ovatus*	Japan	987	D82852

25	*B. japonica*	NT112	*Ixodes ovatus*	Japan	987	D82853
26	*B. lusitaniae*	PotiB1	*Ixodes ricinus*	Portugal	456	DQ111035
27	*B. turdi*	Yh501	*Ixodes turdus*	Japan	876	D85071
28	*B. valaisiana*	OM95/01	*Mus caroli*	Japan	600	AB091706
29	*B. valaisiana*	QSYSP4	*Haemaphysalis longicornis*	China	913	EU135604
30	*Borrelia* sp.	B	*Ixodes pararicinus*	Uruguay	459	JX082312
31	*Borrelia* sp.	Pampa	*Ixodes longiscutatus*	Brazil	318	KY657353

Table S5. *Outer surface protein A* gene of *Borrelia* species included in the phylogenetic analysis summarized according to species, strain or isolate, length (bp), and GenBank accession number.

	Species	Strain or isolate	Host	Country	length (bp)	GenBank accession no.
1	*B. garinii*	DT-42	*Ixodes nipponensis*	Korea	313	KU848761
2	*B. afzelii*	WDT-43	Water deer tick	Korea	310	KU848759
3	*B. afzelii*	214	*Ixodes ricinus*	Luxembourg	779	GU826938
4	*B. bavariensis*	2_10A_263	*Aedes vexans*	Germany	225	ln650625
5	*B. bavariensis*	lp54	Tick	Slovakia	700	JX274591
6	*B. bavariensis*	Ptrob	Human	Germany	282	GQ178234
7	*B. bavariensis*	CSF1411	Human	Czech Republic	302	KT934525
8	*B. bavariensis*	CSF1851	Human	Czech Republic	305	KT934526
9	*B. burgorferi*	297	Human	USA	822	X85442
10	*B. burgorferi*	IPT23	*Ixodes ricinus*	France	282	FJ546645
11	*B. garinii*	IO-TP-TW	*Ixodes ovatus* on Cat	Taiwan	315	KM397123
12	*B. garinii*	Strain: Pbi	Human	Germany	822	X80257
13	*B. garinii*	Mng 1602	*Ixodes persulcatus*	Mongolia	822	DQ479282
14	*B. garinii*	T-MDJ	*Dermacentor* sp.	China	802	HM007278
15	*B. garinii*	39022	*Ixodes persulcatus*	Russia	819	EU635991
16	*B. garinii*	Tom 7105	*Ixodes persulcatus*	Russia	822	DQ479280
17	*B. garinii*	1205	*Ixodes ricinus*	Luxembourg	670	GU826980
18	*B. garinii*	KKU5	N/A	Japan	669	AB009862
19	*B. garinii*	IPT165	*Ixodes ricinus*	France	285	FJ546618
20	*B. japonica*	Strain: H014	*Ixodes ovatus*	Japan	822	Y10893
21	*B. japonica*	IKA2	*Ixodes ricinus*	Belgium	822	Y10892
22	*B. turdi*	Ya501	*Ixodes turdus*	Japan	953	AB016975
23	*B. valaisiana*	M49	*Ixodes ricinus*	Netherlands	825	AF095945

B. valaisiana	VS116	*Ixodes nipponensis*	Korea	945	AB016979
